# Surgical interventions for intractable migraine: a systematic review and meta-analysis

**DOI:** 10.1097/JS9.0000000000001480

**Published:** 2024-04-15

**Authors:** Tahani Alrahbeni, Ahmed Mahal, Anas Alkhouri, Hadil F. Alotaibi, Vineet Rajagopal, Ashish Behera, Khalid AL-Mugheed, Mahalaqua N. Khatib, Shilpa Gaidhane, Quazi S. Zahiruddin, Muhammed Shabil, Ganesh Bushi, Sarvesh Rustagi, Neelima Kukreti, Prakasini Satapathy, Ranjan K. Mohapatra, Arkadiusz Dziedzic, Bijaya K. Padhi

**Affiliations:** a Molecular Toxicology and Genetics, Riyadh Elm University; b Department of Medical Biochemical Analysis, College of Health Technology, Cihan University-Erbil, Erbil, Kurdistan Region, Iraq; c College of Pharmacy, Cihan University-Erbil, Erbil, Kurdistan Region, Iraq; d Department of Pharmaceutical Sciences, College of Pharmacy, Princess Nourah Bint Abdul Rahman University, Riyadh Saudi Arabia; e Department of Community Medicine and School of Public Health, Postgraduate Institute of Medical Education and Research, Chandigarh, India; f Department of Internal Medicine, Postgraduate Institute of Medical Education and Research, Chandigarh, India; g Adult Health Nursing and Critical Care. Riyadh Elm University, Saudi Arabia; h Division of Evidence Synthesis, Global Consortium of Public Health and Research, Datta Meghe Institute of Higher Education, Wardha, India; i One Health Centre (COHERD), Jawaharlal Nehru Medical College, Datta Meghe Institute of Higher Education, Wardha, India; j Global Health Academy, Division of Evidence Synthesis, School of Epidemiology and Public Health and Research, Jawaharlal Nehru Medical College, Datta Meghe Institute of Higher education and Research, Wardha. India; k EvidenceSynthesis Lab, Kolkata, India; l Global Center for Evidence Synthesis, Chandigarh, India; m School of Applied and Life Sciences, Uttaranchal University, Dehradun, Uttarakhand, India; n School of Pharmacy, Graphic Era Hill University, Dehradun, India; o Center for Global Health Research, Saveetha Medical College and Hospital, Saveetha Institute of Medical and Technical Sciences, Saveetha University, Chennai, India; p Medical Laboratories Techniques Department, AL-Mustaqbal University, Hillah, Babil, Iraq; q Department of Chemistry, Government College of Engineering, Keonjhar, Odisha, India; r Department of Conservative Dentistry with Endodontics, Medical University of Silesia, Katowice, Poland

**Keywords:** intractable, migraine, nerve decompression, occipital nerve stimulation, surgery

## Abstract

**Background:**

Migraine affects ~14–15% of the global population, contributing to nearly 5% of the world’s health burden. When drug treatments prove ineffective for intractable migraines, highly specific surgical interventions emerge as potential solutions. The authors aimed to analyze surgical approaches for these refractory or intractable migraines through a systematic review and meta-analysis.

**Methods:**

The authors conducted a literature search across databases such as PubMed, Scopus, Web of Science, and Embase, focusing on studies related to migraines and surgical outcomes. The authors considered clinical trials or observational studies that included any surgical intervention for refractory or intractable migraines, emphasizing key outcomes such as reductions in migraine intensity, Migraine Disability Assessment scores (MIDAS), and 50% Migraine Headache Index (MHI) reduction rates. Statistical analyses were performed using R version 4.3.

**Results:**

Eleven studies were included in the systematic review. A meta-analysis of four studies involving overall 95 patients showed a significant reduction in mean migraine intensity scores using ONS (−2.27, 95% CI: −3.92 to −0.63, *P*=0.021). Three studies with 85 patients showed an average MIDAS score reduction of −52.3, though this was not statistically significant (95% CI: −136.85 to 32.19, *P*=0.116). Two additional studies corroborated these reductions in MIDAS scores. Nerve decompression surgery showed a substantial decrease in the average migraine intensity (from 8.31 down to 4.06). Median MIDAS score dropped from 57 to 20. Two studies indicated a success rate of 40 and 82%, respectively, in achieving a 50% reduction in the migraine MHI through nerve decompression. Findings from two studies suggest that septorhinoplasty and sinus surgery effectively decrease migraine intensity scores.

**Conclusion:**

The existing evidence emphasizes the potential advantages of surgical interventions as a promising approach to managing intractable or refractory migraines. However, robust and comprehensive research is crucial to refine and solidify the efficacy of these surgical methods, aiming for widespread benefits for patients, considering cost-effectiveness factors.

## Introduction

Highlights

- Surgical approaches, notably ONS, significantly reduced migraine intensity.
- Observed reductions in MIDAS scores were notable but not statistically significant.
- Nerve decompression yielded substantial decreases in migraine intensity and had varying success rates in reducing migraine MHI.
- Both septorhinoplasty and sinus surgery were effective in mitigating migraine intensity.
- The findings emphasize the potential of surgical interventions for intractable migraines, highlighting the need for further refinement and research.

A migraine is an episodic headache with specific traits like heightened sensitivity to light, sound, or motion. Moreover, it can appear as a recurring headache syndrome linked with a range of neurological symptoms^1^. This includes associated conditions like cyclic vomiting, somnambulism, abdominal migraine, benign paroxysmal torticollis, benign paroxysmal vertigo, and confusional migraine. These various syndromes have distinct clinical features, durations, and prevalence rates^2^. The nature of migraine follows a cyclic pattern involving multiple phases: the premonitory phase, fleeting neurological symptoms referred to as migraine aura, an intense headache episode, and the postdrome phase^3^. Beyond its physical toll, migraine substantially burdens different aspects of an individual’s life, encompassing financial status, family relationships, and participation in work or studies^4^. At present, the prevalence of migraine stands at ~14–15%. This condition contributes to around 4.9% of the overall burden of health issues experienced by the global population, as measured in years lived with disability (YLDs)^5^. Migraines can be categorized into two distinct groups: resistant migraines, characterized by a lack of response to at least three different classes of migraine preventatives, accompanied by a minimum of eight incapacitating headache days each month for a continuous period of 3 months without any improvement; and refractory migraines, where all available preventative treatments have proven ineffective, and individuals experience a minimum of eight debilitating headache days per month for a continuous duration of 6 months^6^. Ordinary migraine episodes can often be alleviated with interventions like triptan medications and pain relievers. However, in the case of intractable migraines, these treatments may not yield favorable responses.

For individuals who have not experienced positive outcomes from alternative treatments, surgical intervention becomes a viable option for addressing intractable migraines^7^. The peripheral theory of migraines finds support in the pain relief experienced by many patients through treatments like Botulinum toxins or local nerve blocks^7^. Surgical approaches for migraines could involve decompressing one or more nerves, akin to the treatment methodologies employed for conditions like cubital tunnel syndrome, carpal tunnel syndrome, or thoracic outlet syndrome^8^. Surgical approaches for managing migraines encompass a diverse range of methods, including procedures such as peripheral nerve decompression via myectomy or foraminotomy, nerve excision, artery resection, and others, reflecting their heterogeneous nature^7^. Occipital nerve Stimulation (ONS) is another procedure with help to alleviate migraine headache^9^. It involves implanting a small electrical device near the occipital nerve at the skull’s base. This device delivers continuous electrical impulses to the nerve, which is believed to modulate pain signals, thereby reducing the frequency and severity of migraine attacks^9^.

Numerous systematic reviews have explored the field of invasive, surgical treatments for migraines, reporting varying results for different types of surgical procedures^10–13^. Previous reviews have shown that nerve decompression surgery can be beneficial, yet not all patients have experienced significant reductions in migraine frequency or intensity. Notably, no previous systematic reviews have focused solely on intractable migraines. Understanding the efficacy of surgical techniques specifically for intractable migraine is crucial. Findings across studies have been mixed, revealing diverse degrees of evidence regarding the effectiveness of such treatments. Our study aims to address these gaps by conducting a comprehensive systematic review and meta-analysis on surgical interventions for intractable or refractory migraines. By focusing exclusively on this particularly challenging subset of migraine patients, who suffer from severe, debilitating pain and significant reductions in quality of life, we underscore the urgency and novelty of our research. The recent emergence of studies centered on surgical approaches for migraine treatment further underlines the need for our study to validate the effectiveness of these interventions in reducing migraine symptoms, specifically addressing the needs of patients who often struggle the most.

## Methods

The systematic review followed the Preferred Reporting Items for Systematic Reviews and Meta-Analyses (PRISMA) guidelines^14,15^, as detailed in Table S1 (Supplemental Digital Content 2, http://links.lww.com/JS9/C406). This study has been registered in PROSPERO.

### Inclusion and exclusion criteria

This systematic review aimed to assess the efficacy of selected surgical procedures specifically for intractable migraines. We focused on migraines that are refractory or intractable, meaning they do not respond to drug therapy. Our analysis was limited to surgical interventions, such as nerve decompression or ONS. We excluded remote interventions without surgery. Both observational studies (cohort and case–control) and clinical trials were considered for inclusion. Key outcomes of interest included reductions in migraine intensity, changes in Migraine Headache Index (MHI) and Migraine Disability Assessment (MIDAS) scores, the proportion of participants experiencing at least a 50% reduction in symptoms, and reductions in migraine frequency.

We excluded narrative reviews, protocols, unpublished reports, editorials, clinical case reports, commentaries, and abstracts as they did not align with the focus of our study. Our review encompassed only preprints and articles published in English, with no limitations on geographical location or research setting. For more information on the inclusion criteria, refer to Table S2 (Supplemental Digital Content 2, http://links.lww.com/JS9/C406).

### Search strategy and screening

We conducted a literature search across multiple databases, including PubMed, Web of Science, Scopus, and Embase, from inception until 05 August 2023. The search strategy combined keywords, MeSH terms, and synonyms pertinent to migraines, surgery, and associated outcomes. We specifically focused on publications in English, with no restrictions concerning the year of publication. Please refer to Table S3 (Supplemental Digital Content 2, http://links.lww.com/JS9/C406) for further details.

We utilized AutoLit, Nested Knowledge, a semi-automated software platform for removing duplicates, screening, and extraction processes. Upon retrieval of search results, an initial screening of the identified articles was undertaken by two independent researchers. Both researchers autonomously assessed the titles and abstracts to exclude articles not pertinent to the study’s objectives. After this preliminary screening, a detailed evaluation of the full-text articles was conducted to ascertain their relevance and eligibility for inclusion in this systematic review. In instances of discrepancies or divergences in opinion between the two primary researchers, a third senior researcher was consulted to arbitrate and provide a definitive inclusion decision.

### Data extraction and quality assessment

Two investigators undertook the task of data extraction from the selected studies. The extracted data included the first author’s name, the country of the study’s origin, the year of publication, participants’ age, demographic specifics, population type, total sample size, type of the intervention, and each pertinent outcome such as reduction in intensity score, rate of patients who got 50% reduction in migraine headache and MIDAS scores.

We assessed the quality of included RCTs using the Cochrane Risk of Bias 2 (RoB 2) tool, which examines several bias domains to ensure study reliability^16^. This involves a detailed examination of five critical bias domains: bias arising from the randomization process, bias due to deviations from intended interventions, bias due to missing outcome data, bias in measurement of the outcome, and bias in selection of the reported result. Each domain is assessed to determine the risk of bias as ‘low’, ‘some concerns’, or ‘high’, based on specific criteria outlined by the RoB 2 tool. The overall risk of bias in a study is determined by the highest risk level found in any domain. If any domain is judged high, the study has a high risk of bias. If all domains are low, the study has a low risk of bias. If any domain has some concerns without any high risks, the study is considered to have some concerns overall. For observational studies, we applied the Newcastle–Ottawa Scale (NOS) to evaluate their quality based on three domains: selection, comparability, and outcome measurement. High-quality studies score 7–9, meeting most criteria across domains; moderate-quality studies score 4–6, meeting some criteria but with flaws; low-quality studies score 0–3, failing to meet several criteria.

### Statistical analysis

We utilized a random-effects model to determine the combined effect sizes to address potential research discrepancies. DerSimonian–Laird (DL) estimator was used for the meta-anlaysis. This method acknowledges the natural differences in the studies and offers a more reliable estimate of the overall effect^15^. The variability in outcomes across studies, termed heterogeneity, was assessed using *I* ^2^ and tau-squared metrics^17,18^. *I*² values can range from 0 to 100%, with higher values indicating greater heterogeneity. Heterogeneity is classified as high (*I*²>50%), moderate (*I*²=26–50%), or low (*I*²<25%)^19,20^. We set a specific threshold in advance to gauge the statistical relevance of the detected heterogeneity. The 95% prediction interval is a statistical measure used to estimate the range within which future observations are expected to fall with a 95% confidence level^21,22^. Generally, a *P*-value of under 0.05 is deemed statistically relevant. The tau-squared value is derived from the maximum likelihood estimation technique^23^. All our statistical analyses were executed with the R software, version 4.3.0^24^.

### Certainty of evidence

We used the grading of Recommendations, Assessment, Development, and Evaluations (GRADE) approach for assessing the certainty of evidence^25^. The GRADE approach assesses the quality of evidence across studies in a systematic review, classifying it into four levels: high, moderate, low, or very low, based on the risk of bias, inconsistency, indirectness, imprecision, and publication bias. Each outcome for each intervention was assessed for certainty of evidence by using GRADEpro.

## Results

### Literature search

**Figure 1:**
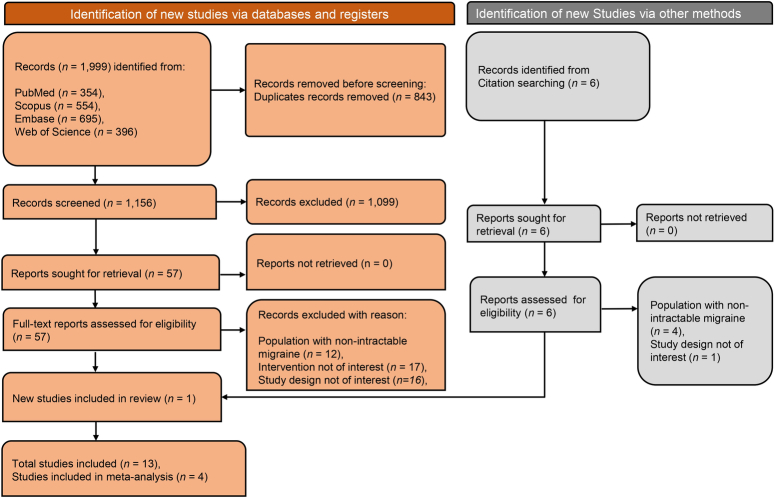
PRISMA flow diagram depicting screening and selection of studies.

A comprehensive literature search was conducted across various databases to identify eligible studies. Figure 1 illustrates the process of identifying and selecting relevant studies. Initially, 1999 articles were retrieved from PubMed, Scopus, Embase, and Web of Science databases. Among these, 843 duplicates were eliminated, and the remaining articles underwent primary screening. During the primary screening, which involved assessing titles and abstracts, 1099 articles were excluded for various reasons, leaving 57 articles to proceed to the full-text screening phase. Following full-text screening, only ten studies satisfied the inclusion criteria. Additionally, a cross-reference search was performed to uncover additional studies, identifying six relevant articles, of which one met the inclusion criteria. A total of 13 were included in the study, of which 10 were observational studies, and the remaining three were randomized controlled trials (RCTs).

### Characteristics of included studies

The characteristics of the included studies in the review are given in Table 1. A total of 519 subjects were involved in the studies. The studies were conducted in different regions globally. Specifically, eight were conducted in the USA^9,26–32^, two in Iran^33,34^, and one each in Spain^35^, UK^36^, and Italy^37^. Various types of surgeries/interventions were performed across studies for the treatment of intractable or refractory migraine. Nerve Depression in three studies^26,27,33^, Functional Endoscopic Sinus Surgery^32^, Septorhinoplasty^34^ in each study and seven studies performed ONS^9,29–31,35–37^. Most studies were conducted in the USA and focused specifically on Nerve Decompression surgery. Among the three RCTs, one showed a high risk of bias^30^, and the other two had some concerns regarding the risk of bias^31,37^. The observational studies were assessed to be of moderate quality overall (Table S4, Supplemental Digital Content 2, http://links.lww.com/JS9/C406).

**Table 1:** Characteristics of included studies.

								Outcomes					
								Migraine intensity		MIDAS			
Study	Country	Study design	Population	Age (mean)	Male %	Sample size	Type of surgery/intervention	Baseline mean (SD)	Final mean (SD)	Baseline mean (SD)	Final mean (SD)	50% MHI reduction (%)	Follow-up duration
Albano *et al.*, 2023^28^	USA	Prospective observational study	Chronic migraine patients failed drug therapy	43	18	34	Decompression or neurectomy	NA	NA	57 (87) Median	20 (59) Median	NA	20.7 mo
Behin *et al.*, 2005^32^	USA	Prospective observational study	Migraine patients failed at least three drugs	45	NA	21	Functional Endoscopic Sinus Surgery	7.8 (1.5)	3.6 (3.7)	NA	NA	NA	62 mo
Dirnberger *et al.*, 2004^27^	USA	Prospective observational study	Migraine patients not responding to drug therapy	NA	NA	60	Nerve Decompression	NA	NA	NA	NA	40	Six months
Gfrerer *et al.*, 2019^26^	USA	Prospective observational study	Refractory migraine Patients	45	14	85	Nerve Decompression	NA	NA	NA	NA	82	12 mo
Ghazisaidi *et al.*, 2012^34^	Iran	Prospective observational study	Patients with a refractory migraine and deviated nose	NA	NA	24	Septorhinoplasty	8.9 (8–10)	0.72 (0–3)	NA	NA	NA	31 mo
Hann *et al.*, 2013^29^	USA	Prospective observational study	Polypharmacological therapy failed chronic migraine patients	41.5	21	14	Occipital nerve stimulation	7.32 (2.4)^a^	3.4 (2.3)	NA	NA	NA	31 mo
Mekhail *et al.*, 2016^38^	USA	Randomized, Double-blind, Controlled trial	Chronic migraine patients failed drug therapy	44.6	25	13	Occipital nerve stimulation	7.29 (1.20)	5.14 (2.25)	168 (55.36)	86.43 (73.58)	NA	13 mo
Miller *et al.*, 2017^39^	UK	Prospective cohort study	Intractable chronic migraine	47.7	24	53	Occipital nerve stimulation	6 (1.71)	4.66 (2.59)	154.91 (84.03)	134.28 (92.7)	NA	42 mo
Omranifard *et al.*, 2004^40^	Iran	Prospective, randomized, controlled trial	Migraine patients not controlled by drug treatment	42.2	12	25	Nerve Decompression	8.31 (0.28)	4.06 (0.18)	NA	NA	NA	12 mo
Rodrigo *et al.*, 2017^35^	Spain	Prospective observational study	Chronic migraine patients with poor response to drug	46.9	NA	35	Occipital nerve stimulation	9.1 (0.64)	Decreased by 4.9 (2.0)	NA	NA	NA	112.8 mo
Schwedt *et al.*, 2007^9^	USA	Retrospective observational study	Medically intractable migraine	39	20	15	Occipital nerve stimulation	7.1 (1.3)	4.7 (2.2)	178 (80)	109 (92)	NA	19 mo
Serra *et al.*, 2012^37^	Italy	Randomized cross-over study	Chronic refractory migraine	46	34	29	Occipital nerve stimulation	NA	NA	79 (30–135)	10 (0–20)	NA	12 mo
Silberstein *et al.*, 2012^31^	USA	Prospective, randomized, controlled trial	Refractory chronic migraine Patients	45	22.9	105	Occipital nerve stimulation	5.99 (1.68)	NA	158.4 (76.8)	Reduced by 64.6 points	NA	18.3 mo

a Calculated by adding mean follow-up score and mean reduction score.

NA, Not available.

### Occipital nerve stimulation

**Figure 2:**
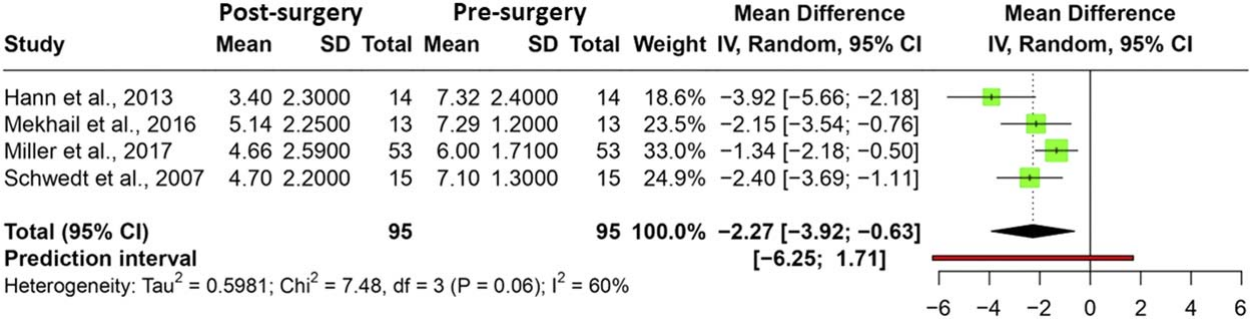
Forest plot depicting the mean difference in migraine intensity presurgery and postsurgery.

Seven studies analyzed the efficacy of ONS for intractable or refractory migraine. The migraine intensity score (on a scale of 0–10) was reported in four studies for ONS surgery. The meta-analysis, which included 95 patients, showed a significant decrease in the mean migraine intensity score of −2.27 (95% CI: −3.92; −0.63) (*P*=0.021), with a prediction interval of −6.25 to 1.71. A heterogeneity with an *I* ^2^ value of 60% was observed (*P*=0.06). Figure 2 illustrates a forest plot of the meta-analysis. Rodrigo *et al*. also reported a decrease in mean migraine intensity scores by 4.9 points from 9.1 in 35 patients. The certainty of the evidence was found to be very low for reducing migraine intensity with ONS due to imprecision (Table S5, Supplemental Digital Content 2, http://links.lww.com/JS9/C406).

**Figure 3:**
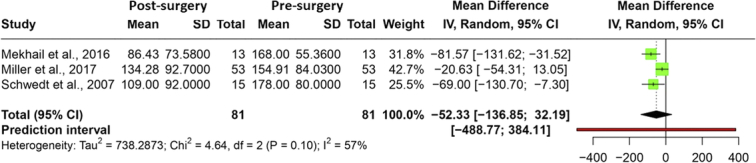
Forest plot depicting the mean difference in MIDAS score presurgery and postsurgery.

Five studies reported changes in MIDAS scores with ONS. Among these, three studies reported baseline and final MIDAS scores as mean and SD, including 81 patients. A reduction in the mean MIDAS score of −52.3 (95% CI: −136.85–32.19) (*P*=0.116) with a prediction interval of −488.7 to 384.1 was observed. A moderate heterogeneity with an *I* ^2^ value of 57% was observed (*P*=0.10). Figure 3 illustrates a forest plot of the meta-analysis. Serra *et al*. reported baseline and final MIDAS scores in mean and range for 34 patients who underwent ONS. The baseline mean MIDAS score was reduced from 79 (30–135) to 10 (0–20). Similarly, Silberstein *et al*. reported a reduction in MIDAS by 64.6 points post-ONS surgery in 105 patients. The certainty of the evidence was found to be moderate for the reduction in MIDAS with ONS due to imprecision (Table S5, Supplemental Digital Content 2, http://links.lww.com/JS9/C406).

### Nerve decompression surgery

Three studies were available for nerve decompression surgery for intractable migraine. Omranifard *et al*. found a reduction in the mean migraine intensity scale from 8.31 (0.28) to 4.06 (0.18) postsurgery by nerve decompression. The certainty of the evidence was found to be low for the reduction of migraine intensity with nerve decompression due to imprecision (Table S6, Supplemental Digital Content 2, http://links.lww.com/JS9/C406). Albano *et al*. found a median MIDAS reduction from 57 to 20 scores after surgery. The certainty of the evidence was very low for the reduction in MIDAS score with nerve decompression due to imprecision (Table S6, Supplemental Digital Content 2, http://links.lww.com/JS9/C406).

Two studies reported a 50% reduction in MHI with nerve decompression surgery. Dirnberger *et al*. reported that 40% of the participants experienced a 50% reduction in MHI with surgery. Similarly, Grefer *et al*. found that 82% of the participants who underwent surgery experienced a 50% reduction in MHI. The certainty of the evidence was found to be very low for the 50% reduction in MHI with nerve decompression due to imprecision (Table S6, Supplemental Digital Content 2, http://links.lww.com/JS9/C406).

### Septorhinoplasty and functional endoscopic sinus surgery

Two studies were available which evaluated the efficacy of nose-related surgeries for migraine headaches. Behin *et al*. conducted a study on the efficacy of functional sinus surgery among 21 patients with intractable migraines and found a mean reduction in the Migraine Intensity Scale from 7.8 (1.5) to 3.6 (3.7). Septorhinoplasty was assessed by Ghazisaidi *et al*. among 24 patients, and they found a mean reduction in migraine intensity from 8.9 to 0.72. The certainty of evidence was very low for migraine intensity reduction due to imprecision (Table S7, Supplemental Digital Content 2, http://links.lww.com/JS9/C406).

### Publication bias

Due to the limited number of studies in the meta-analysis, we could not perform the publication bias assessment statistically.

## Discussion

This systematic review and meta-analysis showed a significant postsurgical decrease in migraine intensity MIDAS and the proportion of participants with 50% MHI. Nerve decompression and ONS, the most studied and clinically proven techniques, yielded promising results. The considerable mean difference postsurgery indicates potential therapeutic benefits for patients suffering from intractable migraines. These findings provide a substantial contribution to the field of migraine management, particularly in addressing the challenges of intractable or refractory migraines. The novelty of this research lies in its specific focus on the effectiveness of surgical interventions for this subset of migraines. This area has previously garnered skepticism within the medical community. A thorough meta-analysis provides empirical evidence supporting the efficacy of surgical approaches like nerve decompression and ONS, showcasing their potential to reduce migraine intensity and MIDAS scores significantly. This is particularly noteworthy as it offers a new direction for treatment where conventional methods have failed.

Although our study is the first meta-analysis to evaluate surgery for intractable migraine in specific, several other systematic reviews and meta-analyses have already addressed surgical interventions for certain types of migraines. To facilitate and quantify analysis, we established specific criteria for intractable or refractory migraine. For instance, the study by Elwahary *et al*.^11^ found that migraine surgery significantly reduced migraine outcomes (duration, intensity, and frequency) and led to an overall decrease in the MHI. Their meta-analysis showed enhanced results postmigraine surgery, albeit with some variability. This inconsistency arises from the inclusion of diverse surgical techniques, differences in study parameters, and the evolving landscape of migraine surgery. While past skepticism existed, the current literature supports the surgery’s efficacy. However, safety concerns persist, with the American Headache Society emphasizing the need for more evidence^41^. Their study analyzed complication rates and found that, although over a third of the 1645 patients experienced complications, most were minor. Similarly, another systematic review by Henriques *et al*.^13^ presented robust clinical data from high-impact journals supporting extracranial surgical treatment’s safety and efficacy for migraine headaches.

The predominance of US-based research in our systematic review and meta-analysis may reflect the advanced state of medical research and surgical innovation within the country, particularly in the field of migraine treatment. However, this concentration also suggests a need for caution when applying these results globally. The variability in healthcare systems, access to surgical interventions, and patient populations across different regions could influence the effectiveness and feasibility of these surgical treatments for intractable migraines. Therefore, while our findings indicate promising therapeutic benefits of surgical interventions such as nerve decompression and ONS, the potential impact of regional differences on treatment outcomes cannot be overlooked. Further research involving diverse geographical locations is crucial to validate the efficacy of these interventions in a broader context and to understand any variations in treatment response among different populations. This aspect underscores the importance of including studies from a wider range of countries in future research to ensure the findings are applicable and beneficial to migraine sufferers worldwide.

The findings from our meta-analysis indicate the potential therapeutic benefits of surgical interventions for intractable or refractory migraines. Clinicians may consider these surgical options viable alternatives for patients who have not responded to conventional treatment. The consistent outcomes across various surgical techniques suggest that there is not a one-size-fits-all approach, allowing for tailored interventions based on individual patient needs and identified triggers. As the field evolves, future research should further prioritize large-scale, multicenter randomized controlled trials to validate the efficacy and safety of these surgical interventions. Additionally, long-term follow-up studies are crucial to assessing surgical outcomes’ sustainability and potential complications. Given the skepticism in parts of the medical community, especially among neurologists, bridging the knowledge gap through interdisciplinary collaborations is imperative, ensuring that the benefits of migraine surgery reach a broader audience. Lastly, with the emerging understanding of peripheral nerve irritation’s role in migraines, research should progress deep into the mechanisms underlying surgical success, potentially paving the way for innovative, less invasive interventions.

Our study has some limitations that warrant consideration. Firstly, we restricted our inclusion criteria to articles published solely in English, which may have introduced a language bias. Secondly, the outcomes, such as migraine intensity, MHI, and MIDAS, were based on patient-reported outcomes. Such self-reported measures are inherently subjective and can vary based on individual perceptions, potentially introducing bias into research findings. Quantitative analysis was only possible for some of the studies due to the limited number of studies available and the need to stratify them by the type of surgery and the nature of the reported outcomes. We acknowledge a significant limitation in our study’s design, which is the reliance on preintervention and postintervention values without the comparison to a control group. This methodological choice restricts our ability to definitively attribute the observed changes solely to the surgical intervention, as other potential factors or confounding variables could also influence the outcomes. The absence of a direct statistical comparison between intervention and control groups limits the strength of our conclusions regarding the efficacy of the surgical treatments for intractable or refractory migraines. The limited number of trials included in our analysis, with only three clinical trials incorporated, underscores the need for more comprehensive and robust research.

## Conclusion

This study indicates the clinical potential of surgical interventions as a promising avenue for managing intractable and/or refractory migraines. As the medical community continues to explore this therapeutic approach, interdisciplinary collaboration and extensive research remain vital for fine-tuning and substantiating the effectiveness of dedicated surgical procedures, ultimately leading to broader patient benefits.

## Ethical approval

Not required.

## Informed consent statement

Not applicable.

## Source of funding

None.

## Author contribution

B.K.P., T.A., A.M., A.A., H.F.A., and V.R.: concept; M.S., G.B., and B.K.P.: data acquisition; A.B., K.A., M.N.K., S.G., and Q.S.Z.: data analysis; B.K.P., T.A., A.M., A.A., H.F.A., V.R., A.B., K.A., M.N.K., and S.G.: drafting of manuscript; M.N.K., S.G., Q.S.Z., M.S., G.B., S.R., N.K., P.S., R.K.M., and A.D.: critical analysis and reviewing; B.K.P., T.A., A.M., A.A., H.F.A., V.R., A.B., K.A., M.N.K., S.G., Q.S.Z., M.S., G.B., S.R., N.K., P.S., R.K.M., and A.D.: approval for final version.

## Conflicts of interest disclosure

The authors declare no conflicts of interest.

## Research registration unique identifying number (UIN)

PROSPERO: CRD42023464281.

## Guarantor

Bijaya Kumar Padhi.

## Data availability statement

Documents containing all extracted data are available in the manuscript and the accompanying Supplementary Material (Supplemental Digital Content 1, http://links.lww.com/JS9/C407).

Data are available with authors and available on request. Some data are available in supplementary material file.

## Provenance and peer review

Invited.

## Institutional review board statement

Not applicable.

## Supplementary Material

SUPPLEMENTARY MATERIAL
