# Supplementary material for: Surgical interventions for intractable migraine: a systematic review and meta-analysis
Source: Int J Surg. 2024 Apr 15;110(10):6306–13. doi: 10.1097/JS9.0000000000001480 (PMC11486983; doi:10.1097/JS9.0000000000001480)
Supplement: SUPPLEMENTARY MATERIAL [file js9-110-6306-s001.docx]

**Supplementary Materials**

**Table S1.** PRISMA Checklist

**Table S2.** Inclusion and Exclusion criteria

**Table S3.** The adjusted search terms as per searched electronic databases

**Table S4.** Quality assessment using Newcastle-Ottawa Scale and Cochrane RoB2.

**Table S5**. GRADE Summary of evidence for ONS

**Table S6**. GRADE Summary of evidence for nerve decompression

**Table S7.** GRADE Summary of evidence for Septorhinoplasty/ functional endoscopic sinus surgery

## **Table S1.** PRISMA Checklist

| **Section and Topic** | **Item #** | **Checklist item (Prevalence of kidney diseases among the dengue patients: A systematic review and meta-analysis)** | **Location where item is reported** |
| --- | --- | --- | --- |
| **TITLE** | | |  |
| Title | 1 | Identify the report as a systematic review. | 1 |
| **ABSTRACT** | | |  |
| Abstract | 2 | See the PRISMA 2020 for Abstracts checklist. (made as per the Journal guidelines) | 2 |
| **INTRODUCTION** | | |  |
| Rationale | 3 | Describe the rationale for the review in the context of existing knowledge. | 2 |
| Objectives | 4 | Provide an explicit statement of the objective(s) or question(s) the review addresses. | 3 |
| **METHODS** | | |  |
| Eligibility criteria | 5 | Specify the inclusion and exclusion criteria for the review and how studies were grouped for the syntheses. | 3 |
| Information sources | 6 | Specify all databases, registers, websites, organisations, reference lists and other sources searched or consulted to identify studies. Specify the date when each source was last searched or consulted. | 3, Table S2 |
| Search strategy | 7 | Present the full search strategies for all databases, registers and websites, including any filters and limits used. | Table S3 |
| Selection process | 8 | Specify the methods used to decide whether a study met the inclusion criteria of the review, including how many reviewers screened each record and each report retrieved, whether they worked independently, and if applicable, details of automation tools used in the process. | 3 |
| Data collection process | 9 | Specify the methods used to collect data from reports, including how many reviewers collected data from each report, whether they worked independently, any processes for obtaining or confirming data from study investigators, and if applicable, details of automation tools used in the process. | 4 |
| Data items | 10a | List and define all outcomes for which data were sought. Specify whether all results that were compatible with each outcome domain in each study were sought (e.g., for all measures, time points, analyses), and if not, the methods used to decide which results to collect. | 3 |
|  | 10b | List and define all other variables for which data were sought (e.g., participant and intervention characteristics, funding sources). Describe any assumptions made about any missing or unclear information. | 4, Table S4 |
| Study risk of bias assessment | 11 | Specify the methods used to assess risk of bias in the included studies, including details of the tool(s) used, how many reviewers assessed each study and whether they worked independently, and if applicable, details of automation tools used in the process. | Table S5 |
| Effect measures | 12 | Specify for each outcome the effect measure(s) (e.g. risk ratio, mean difference) used in the synthesis or presentation of results. | 5 |
| Synthesis methods | 13a | Describe the processes used to decide which studies were eligible for each synthesis (e.g. tabulating the study intervention characteristics and comparing against the planned groups for each synthesis (item #5)). | Table S2 |
|  | 13b | Describe any methods required to prepare the data for presentation or synthesis, such as handling of missing summary statistics, or data conversions. | NA |
|  | 13c | Describe any methods used to tabulate or visually display results of individual studies and syntheses. |  |
|  | 13d | Describe any methods used to synthesize results and provide a rationale for the choice(s). If meta-analysis was performed, describe the model(s), method(s) to identify the presence and extent of statistical heterogeneity, and software package(s) used. | 5,6, Figure 2 |
|  | 13e | Describe any methods used to explore possible causes of heterogeneity among study results (e.g. subgroup analysis, meta-regression). | 5 |
|  | 13f | Describe any sensitivity analyses conducted to assess robustness of the synthesized results. | 6 |
| Reporting bias assessment | 14 | Describe any methods used to assess risk of bias due to missing results in a synthesis (arising from reporting biases). | NA |
| Certainty assessment | 15 | Describe any methods used to assess certainty (or confidence) in the body of evidence for an outcome. | NA |
| **RESULTS** | | |  |
| Study selection | 16a | Describe the results of the search and selection process, from the number of records identified in the search to the number of studies included in the review, ideally using a flow diagram. | Table S1 |
|  | 16b | Cite studies that might appear to meet the inclusion criteria, but which were excluded, and explain why they were excluded. | Table S1 |
| Study characteristics | 17 | Cite each included study and present its characteristics. | 9,10,11,12,13, Table 1 |
| Risk of bias in studies | 18 | Present assessments of risk of bias for each included study. | Table S4 |
| Results of individual studies | 19 | For all outcomes, present, for each study: (a) summary statistics for each group (where appropriate) and (b) an effect estimate and its precision (e.g. confidence/credible interval), ideally using structured tables or plots. | Table 1, Figure 2 |
| Results of syntheses | 20a | For each synthesis, briefly summarise the characteristics and risk of bias among contributing studies. | 5 |
|  | 20b | Present results of all statistical syntheses conducted. If meta-analysis was done, present for each the summary estimate and its precision (e.g. confidence/credible interval) and measures of statistical heterogeneity. If comparing groups, describe the direction of the effect. | 5, 6, Figure 2 |
|  | 20c | Present results of all investigations of possible causes of heterogeneity among study results. | 6 |
|  | 20d | Present results of all sensitivity analyses conducted to assess the robustness of the synthesized results. | NA |
| Reporting biases | 21 | Present assessments of risk of bias due to missing results (arising from reporting biases) for each synthesis assessed. | NA |
| Certainty of evidence | 22 | Present assessments of certainty (or confidence) in the body of evidence for each outcome assessed. | NA |
| **DISCUSSION** | | |  |
| Discussion | 23a | Provide a general interpretation of the results in the context of other evidence. | 6, 7 |
|  | 23b | Discuss any limitations of the evidence included in the review. | 7 |
|  | 23c | Discuss any limitations of the review processes used. | 7 |
|  | 23d | Discuss implications of the results for practice, policy, and future research. | 7 |
| **OTHER INFORMATION** | | |  |
| Registration and protocol | 24a | Provide registration information for the review, including register name and registration number, or state that the review was not registered. | 3 |
|  | 24b | Indicate where the review protocol can be accessed, or state that a protocol was not prepared. | 3 |
|  | 24c | Describe and explain any amendments to information provided at registration or in the protocol. | NA |
| Support | 25 | Describe sources of financial or non-financial support for the review, and the role of the funders or sponsors in the review. | 8 |
| Competing interests | 26 | Declare any competing interests of review authors. | 8 |
| Availability of data, code and other materials | 27 | Report which of the following are publicly available and where they can be found: template data collection forms; data extracted from included studies; data used for all analyses; analytic code; any other materials used in the review. | 9, Supplementary Materials |

**Table S2.** Inclusion and Exclusion criteria

**Research Question:** Effect of Surgical Interventions for Intractable migraine?

| **Inclusion** | | **Exclusion** |
| --- | --- | --- |
| **Participants** | Patients with intractable or refractory migraine which are not responding to drug therapy | Non migraine headaches, medication use headaches, Migraine which are responding to drug therapy |
| **Intervention** | Any surgical treatment for migraine. | Non-surgical interventions |
| **Outcome** | Migraine intensity, migraine frequency, migraine reduction rate, migraine disability assessment, migraine headache index |  |
| **Study Designs** | RCTs, observational studies, longitudinal studies, retrospective studies, prospective studies, case-control studies, | Qualitative, policy, opinion, case studies, case-reports, reviews, and animal studies |
|  | Geography-Global level  Date of Search- Publish till 15^th^ Aug 2023 |  |
|  | Published articles and preprints data |  |

**Table S3. The adjusted search terms as per searched electronic databases [as of 15.08.2023]**

| **Database** | | | **No** | **Search Query** | | **Results** | |
| --- | --- | --- | --- | --- | --- | --- | --- |
| **PubMed** | | | | | | | |
|  | | | **#1** | **"migraine disorders"[MeSH Terms]** | | **31,886** | |
|  |  |  | **#2** | **Migraine[Title/Abstract] OR migrainosus[Title/Abstract] OR cephalalgia[Title/Abstract] OR hemicrania[Title/Abstract] OR megrim[Title/Abstract] OR "Throbbing headache"[Title/Abstract]** | | **42267** | |
|  |  |  | **#3** | **#1 OR #2** | | **46777** | |
|  |  |  | **#4** | **"surgical*"[Title/Abstract] OR "operation*"[Title/Abstract] OR "invasive procedure*"[Title/Abstract] OR "microsurgical decompression"[Title/Abstract] OR "nerve decompression"[Title/Abstract] OR "nerve surgery"[Title/Abstract] OR "surgical decompression"[Title/Abstract] OR "occipital nerve surgery"[Title/Abstract] OR "surgery"[Title/Abstract] OR "decompression"[Title/Abstract] OR Neurolysis[Title/Abstract] OR Neuromodulation[Title/Abstract] OR foraminotomy[Title/Abstract] OR "nerve stimulation"[Title/Abstract] OR "Trigger site deactivation"[Title/Abstract] OR "muscle resection"[Title/Abstract] OR "nerve resection"[Title/Abstract] OR "myectomy"[Title/Abstract] OR "artery excision"[Title/Abstract]** | | **2,707,478** | |
|  |  |  | **#5** | **"Headache frequency" OR "headache intensity" OR "migraine intensity" OR MHI OR "migraine headache index" OR "migraine elimination" OR "headache duration" OR "migraine duration" OR VAS OR "visual analogue scale" OR duration OR "MH reduction" OR "headache reduction" OR "headache elimination" OR "migraine frequency"** | | **828,590** | |
|  |  |  | **#6** | **#3 AND #4 AND #5Filters: English** | | **354** | |
| **Scopus** | | | | | | | |
|  | | **#1** | **TITLE-ABS-KEY ( migraine OR migrainosus OR cephalalgia OR hemicrania OR megrim OR "throbbing headache" )** | | | **75,011** | |
|  |  | **#2** | **TITLE-ABS-KEY ( “surgical*” OR “operation*” OR “invasive procedure*” OR "microsurgical decompression" OR "nerve decompression" OR "nerve surgery" OR "surgical decompression" OR "occipital nerve surgery" OR "surgery" OR "decompression" OR Neurolysis OR Neuromodulation OR foraminotomy OR “nerve stimulation” OR “Trigger site deactivation” OR “muscle resection” OR “nerve resection” OR “myectomy” OR “artery excision”)** | | | **6,748,797** | |
|  |  | **#3** | **TITLE-ABS-KEY (“Headache frequency” OR “headache intensity” OR “migraine intensity” OR MHI OR “migraine headache index” OR “migraine elimination” OR “headache duration” OR “migraine duration” OR VAS OR “visual analogue scale” OR duration OR “MH reduction” OR “headache reduction” OR “headache elimination” OR “migraine frequency”)** | | | **1,725,308** | |
|  |  | **#4** | **#1 AND #2 AND #3 ( LIMIT-TO ( LANGUAGE , "english" ) ) AND ( LIMIT-TO ( DOCTYPE , "ar" ) )** | | | **554** | |
| **Embase** | | | | |  | |  |
|  |  | | **('migraine'/exp OR migraine OR migraine:ti,ab,kw OR migrainosus:ti,ab,kw OR cephalalgia:ti,ab,kw OR hemicrania:ti,ab,kw OR megrim:ti,ab,kw OR 'throbbing headache':ti,ab,kw) AND ('surgical*':ti,ab,kw OR 'operation*':ti,ab,kw OR 'invasive procedure*':ti,ab,kw OR 'microsurgical decompression':ti,ab,kw OR 'nerve decompression':ti,ab,kw OR 'nerve surgery':ti,ab,kw OR 'surgical decompression':ti,ab,kw OR 'occipital nerve surgery':ti,ab,kw OR 'surgery':ti,ab,kw OR 'decompression':ti,ab,kw OR neurolysis:ti,ab,kw OR neuromodulation:ti,ab,kw OR foraminotomy:ti,ab,kw OR 'nerve stimulation':ti,ab,kw OR 'trigger site deactivation':ti,ab,kw OR 'muscle resection':ti,ab,kw OR 'nerve resection':ti,ab,kw OR 'myectomy':ti,ab,kw OR 'artery excision':ti,ab,kw) AND ('headache frequency':ti,ab,kw OR 'headache intensity':ti,ab,kw OR 'migraine intensity':ti,ab,kw OR mhi:ti,ab,kw OR 'migraine headache index':ti,ab,kw OR 'migraine elimination':ti,ab,kw OR 'headache duration':ti,ab,kw OR 'migraine duration':ti,ab,kw OR vas:ti,ab,kw OR 'visual analogue scale':ti,ab,kw OR duration:ti,ab,kw OR 'mh reduction':ti,ab,kw OR 'headache reduction':ti,ab,kw OR 'headache elimination':ti,ab,kw OR 'migraine frequency':ti,ab,kw)** | | | **714** | |
| **Web of Science** | | | | |  | |  |
|  |  | | **((TS=(Migraine OR migrainosus OR cephalalgia OR hemicrania OR megrim OR “Throbbing headache” )) AND TS=(“surgical*” OR “operation*” OR “invasive procedure*” OR "microsurgical decompression" OR "nerve decompression" OR "nerve surgery" OR "surgical decompression" OR "occipital nerve surgery" OR "surgery" OR "decompression" OR Neurolysis OR Neuromodulation OR foraminotomy OR “nerve stimulation” OR “Trigger site deactivation” OR “muscle resection” OR “nerve resection” OR “myectomy” OR “artery excision”)) AND TS=(“Headache frequency” OR “headache intensity” OR “migraine intensity” OR MHI OR “migraine headache index” OR “migraine elimination” OR “headache duration” OR “migraine duration” OR VAS OR “visual analogue scale” OR duration OR “MH reduction” OR “headache reduction” OR “headache elimination” OR “migraine frequency”)** | | | **396** | |

**Table S4.** Quality assessment using Newcastle-Ottawa Scale and Cochrane RoB2.

| **Newcastle-Ottawa Scale** | | | | | | | | | | |
| --- | --- | --- | --- | --- | --- | --- | --- | --- | --- | --- |
| **Study** | **SELECTION (max 4 points)** | | | | **COMPARABILITY (max 2 points)** | **OUTCOME (max 3 points)** | | | **SCORE (out of 9)** | |
|  | Representativeness of the migraine population | Selection of the Control (non-migraine) | Ascertainment of diagnosis of migraine | Demonstration of the outcome of interest was not present at start of study | Comparability on basis of the design or analysis | Assessment of outcome | Was follow-up long enough for outcomes to occur? | Adequacy of the follow-up |  |  |
| Albano et al., 2023 | 1 | 0 | 1 | 1 | 1 | 1 | 1 | 1 | 7 |  |
| Behin et al., 2005 | 1 | 0 | 1 | 1 | 2 | 1 | 0 | 0 | 6 |  |
| Dirnberger et al., 2004 | 1 | 0 | 1 | 1 | 0 | 1 | 1 | 0 | 5 |  |
| Gfrerer et al., 2019 | 1 | 0 | 1 | 1 | 0 | 0 | 1 | 1 | 5 |  |
| Ghazisaidi et al., 2012 | 1 | 0 | 1 | 1 | 1 | 1 | 0 | 1 | 6 |  |
| Hann et al., 2013 | 1 | 0 | 1 | 1 | 2 | 0 | 1 | 0 | 6 |  |
| Miller et al., 2017 | 1 | 0 | 1 | 1 | 1 | 1 | 1 | 0 | 6 |  |
| Omranifard et al., 2004 | 1 | 1 | 1 | 1 | 1 | 1 | 0 | 1 | 7 |  |
| Rodrigo et al., 2017 | 1 | 0 | 1 | 1 | 0 | 1 | 1 | 1 | 6 |  |
| Schwedt et al., 2007 | 1 | 0 | 1 | 1 | 2 | 1 | 1 | 0 | 7 |  |
|  | | | | | | | | | |  |
| **Cochrane RoB 2 tool for RCTs** | | | | | | | | | |  |
| **Study and author judgement** | **Bias arising from the randomization process** | **Bias due to deviations from intended interventions** | **Bias due to missing outcome data** | **Bias in measurement of the outcome** | **Bias in selection of the reported result** | **Overall** | | | |  |
| Mekhail et al., 2016 | Low risk | Low risk | Low risk | Some concerns | High risk | High risk | | | |  |
| Serra et al., 2012 | Low risk | Some concerns | Low risk | Low risk | Some concerns | Some concerns | | | |  |
| Silberstein et al., 2012 | Low risk | Low risk | Low risk | Some concerns | Some concerns | Some concerns | | | |  |

**Table S5**. GRADE Summary of evidence for ONS

**Author(s):**

**Question:** Occipital nerve stimulation compared to no treatment for Intractable migraine

**Setting:**

**Bibliography:**

| **Certainty assessment** | | | | | | | **№ of patients** | | **Effect** | | **Certainty** | **Importance** |
| --- | --- | --- | --- | --- | --- | --- | --- | --- | --- | --- | --- | --- |
| **№ of studies** | **Study design** | **Risk of bias** | **Inconsistency** | **Indirectness** | **Imprecision** | **Other considerations** | **Occipital nerve stimulation** | **no treatment** | **Relative (95% CI)** | **Absolute (95% CI)** |  |  |
| **Migraine Intensity (assessed with: 0-10 score scale)** | | | | | | | | | | | | |
| 5 | observational studies | not serious | not serious | not serious | serious^a^ | none | 95 | - | - | MD **2.27 Mean score lower** (3.92 lower to 0.63 lower) | ⨁◯◯◯ Very low | CRITICAL  ^d^ |
| **MIDAS (assessed with: Mean MIDAS score)** | | | | | | | | | | | | |
| 5 | randomised trials | not serious | not serious | not serious | serious^b^ | none | 81 | - | - | MD **52.33 Mean MIDAS score lower** (136.85 lower to 32.19 higher) | ⨁⨁⨁◯ Moderate | IMPORTANT  ^c^ |

**CI:** confidence interval; **MD:** mean difference

#### Explanations

a. Confidence interval overlap with clinically insignificant reduction of intensity, Prediction interval overlap with null affect. Sample size is less

b. Confidence interval overlap with null effect, Wide prediction interval and less sample size

**Table S6**. GRADE Summary of evidence for nerve decompression

**Author(s):**

**Question:** Nerve decompression compared to No treatment for Intractable Migraine

**Setting:**

**Bibliography:**

| **Certainty assessment** | | | | | | | **№ of patients** | | **Effect** | | **Certainty** | **Importance** |
| --- | --- | --- | --- | --- | --- | --- | --- | --- | --- | --- | --- | --- |
| **№ of studies** | **Study design** | **Risk of bias** | **Inconsistency** | **Indirectness** | **Imprecision** | **Other considerations** | **Nerve decompression** | **No treatment** | **Relative (95% CI)** | **Absolute (95% CI)** |  |  |
| **Migraine intensity (follow-up: mean 12 months; assessed with: 0-10 Score scale)** | | | | | | | | | | | | |
| 1 | randomised trials | not serious | not serious | not serious | very serious^a^ | none | 25 |  | - | mean **4.25 Mean intensity lower** (0 to 0 ) | ⨁⨁◯◯ Low | CRITICAL |
| **MIDAS (follow-up: mean 20.7 months; assessed with: Median MIDAS score)** | | | | | | | | | | | | |
| 1 | observational studies | not serious | not serious | not serious | very serious^b^ | none | 34 |  | - | median **37 Median MIDAS lower** (0 to 0 ) | ⨁◯◯◯ Very low | IMPORTANT |
| **50% MHI Reduction rate (assessed with: Percentage of patients experiencing 50% MHI reduction)** | | | | | | | | | | | | |
| 2 | observational studies | not serious | not serious | not serious | serious^c^ | none | 40% of patients got 50% migraine intensity reduction out of 60 partients. In another study 82% of patients got 50% migraine reduction out of 85 patients. | | | | ⨁◯◯◯ Very low | IMPORTANT |

**CI:** confidence interval

#### Explanations

a. Only a single trial with 25 Patients for nerve decompression.

b. Only a single observational study with very less sample size

c. Only 2 observational studies with less sample size

**Table S7.** GRADE Summary of evidence for Septorhinoplasty/ functional endoscopic sinus surgery

**Author(s):**

**Question:** Septorhinoplasty/Functional Endoscopic Sinus Surgery compared to No treatment for Intractable migraine

**Setting:**

**Bibliography:**

| **Certainty assessment** | | | | | | | **Impact** | **Certainty** | **Importance** |
| --- | --- | --- | --- | --- | --- | --- | --- | --- | --- |
| **№ of studies** | **Study design** | **Risk of bias** | **Inconsistency** | **Indirectness** | **Imprecision** | **Other considerations** |  |  |  |
| **Migraine intensity (assessed with: 0-10 score scale)** | | | | | | | | | |
| 2 | observational studies | not serious | not serious | not serious | serious^a^ | none | A study on the efficacy of functional sinus surgery among 21 patients with intractable migraines and found a mean reduction in the Migraine Intensity Scale from 7.8 (1.5) to 3.6 (3.7). Septorhinoplasty was assessed by another among 24 patients, and they found a mean reduction in migraine intensity from 8.9 to 0.72. | ⨁◯◯◯ Very low | CRITICAL |

**CI:** confidence interval

#### Explanations

a. Only 2 observational studies with less sample size
