# Supplementary material for: Surgical interventions for intractable migraine: a systematic review and meta-analysis
Source: Int J Surg. 2024 Apr 15;110(10):6306–13. doi: 10.1097/JS9.0000000000001480 (PMC11486983; doi:10.1097/JS9.0000000000001480)
Supplement: SUPPLEMENTARY MATERIAL [file js9-110-6306-s002.docx]

**Supplementary Materials**

**Figure S1.** Funnel plots of migraine intensity

**Figure S2.** Funnel plots of MIDAS


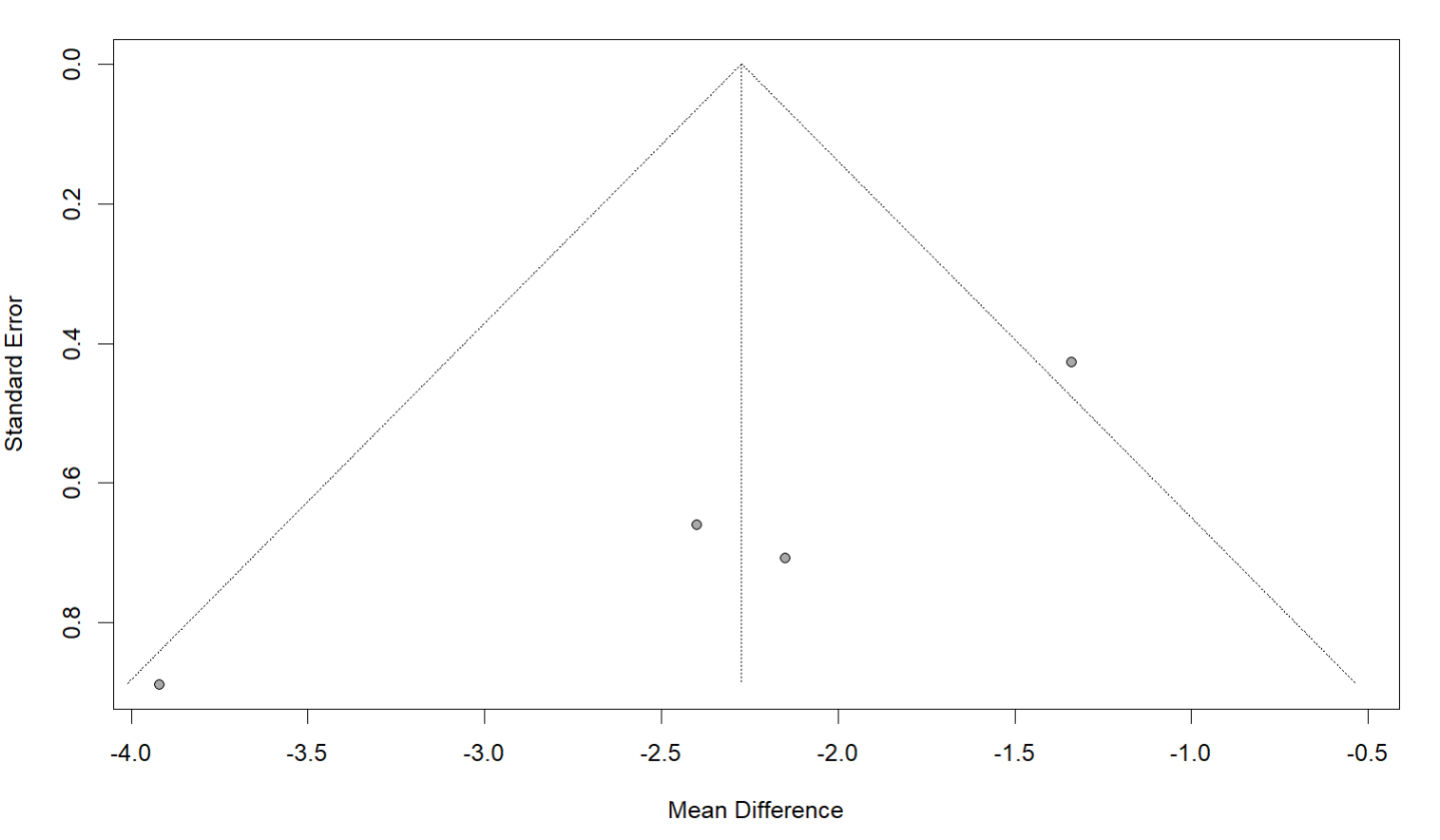


**Figure S1.** Funnel plots of migraine intensity


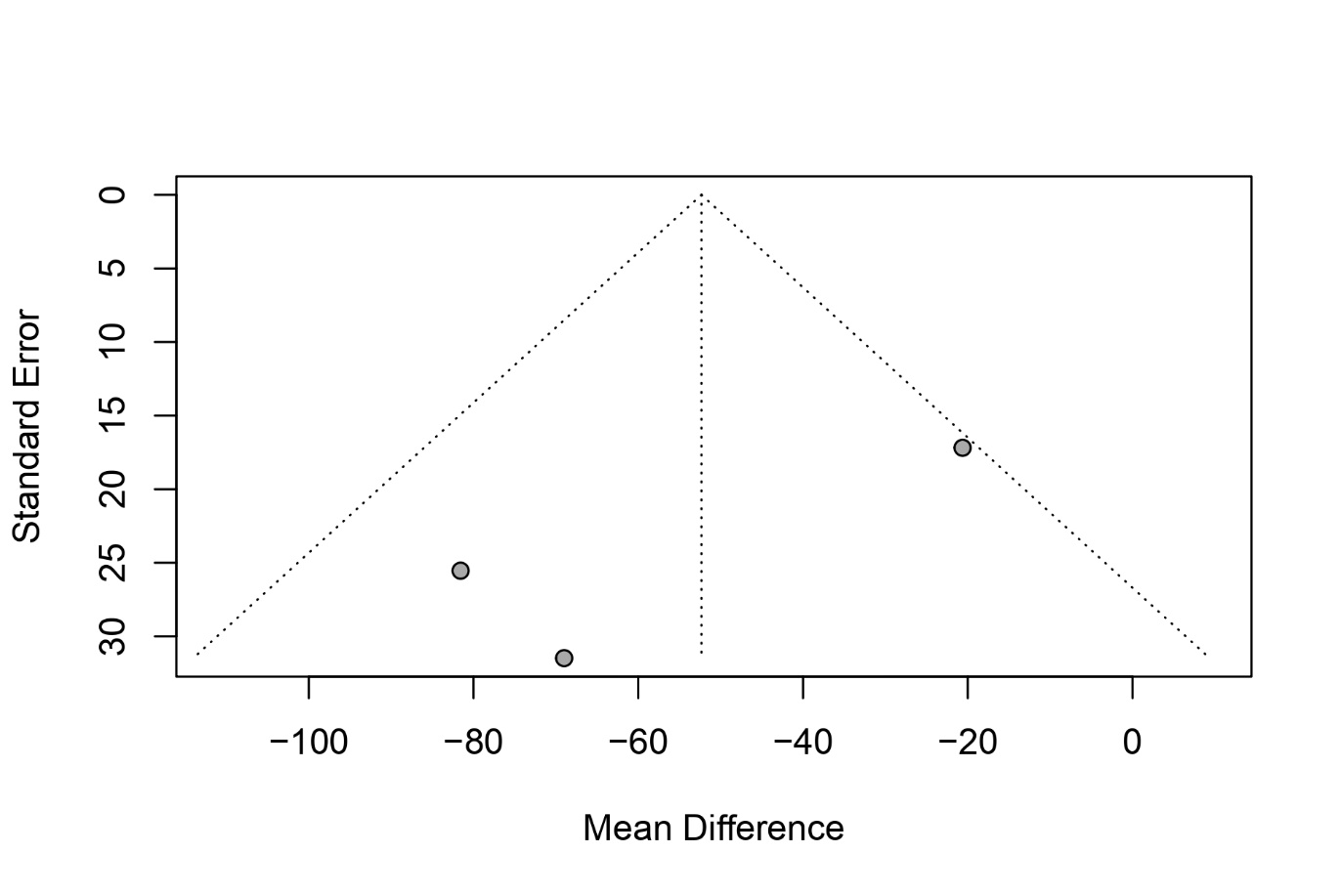


**Figure S2.** Funnel plots of MIDAS
